# Beyond metropolises: artificial intelligence awareness and educational needs among medical students in a developing country

**DOI:** 10.3389/fmed.2025.1645484

**Published:** 2025-09-19

**Authors:** Emre Sanri

**Affiliations:** Department of Pediatrics, Samsun University Faculty of Medicine, Samsun, Türkiye

**Keywords:** artificial intelligence, curriculum development, digital health, medical education, medical students

## Abstract

**Background:**

While artificial intelligence (AI) is rapidly transforming healthcare, medical curricula have not fully adapted to this transformation, particularly in developing countries. This gap is especially pronounced in non-metropolitan regions, where resources and exposure to technology may be more limited. Understanding the perspectives of medical students in these specific contexts is vital for designing effective and equitable educational strategies.

**Objective:**

This study evaluated the knowledge, awareness, attitudes, and educational expectations of students at a newly established Turkish medical school, located in a non-metropolitan city, regarding AI and digital health technologies.

**Methods:**

A descriptive, cross-sectional survey was conducted among first- to fourth-year students at Samsun University Faculty of Medicine. Data were collected from 198 participants, and a stratified random sample of 120 students was selected for analysis. The questionnaire covered demographics, digital literacy, AI knowledge, and attitudes toward its integration into the curriculum.

**Results:**

The majority of students rated their digital competence as moderate (59.2%) or low (19.2%). Interest in technology was highest among first-year students (60%) but declined significantly to 13.3% by the fourth year. Knowledge of AI was generally limited, with only 15.8% reporting a high level of knowledge and 55.0% stating they conducted little or no independent research on AI. Despite these deficiencies, students expressed strong support for AI integration: 78.3% supported AI-assisted interactive tools, 79.2% endorsed personalized learning, and 80% acknowledged the role of AI in clinical decision-making processes. The majority of students (82.5%) advocated for a broader integration of AI, stating that their top priority was its integration into clinical practice (39.2%).

**Conclusion:**

Offering a rare perspective from a non-metropolitan city in a developing country, this study reveals that medical students exhibit high motivation for AI integration despite significant knowledge gaps. This enthusiasm presents a critical opportunity for curriculum reform. There is an urgent need for context-appropriate AI curricula to bridge the gap between student interest and preparedness, thereby empowering the next generation of physicians in diverse settings for the future of digital healthcare.

## 1 Introduction

Artificial intelligence (AI) has emerged as a transformative force in healthcare, influencing various domains including clinical decision support systems, diagnostic imaging, electronic health records, and personalized medicine (1). In this study, we use the term “artificial intelligence (AI)” to refer specifically to computer systems capable of performing tasks that typically require human intelligence, such as learning, reasoning, and decision-making. “Digital health” is used in a broader sense to encompass all technology-enabled healthcare innovations, including AI, telemedicine, mobile health applications, wearable devices, and electronic health records. “Technological innovations” refers to novel tools, platforms, or systems both within and beyond healthcare that enhance learning, practice, or service delivery and may or may not incorporate AI. As AI technologies continue to evolve, it has become imperative for future physicians to acquire a foundational understanding of AI principles, comprehend algorithmic functions and limitations, and develop competencies in data interpretation and ethical reasoning (2).

Despite the increasing emphasis on digital health, recent studies indicate that medical students often possess limited knowledge of AI and have minimal exposure to structured educational content in this field. Many students report having first encountered AI through media or popular discourse, rather than through formal curricula (3, 4). Their attitudes toward AI appear to vary depending on their knowledge levels, ranging from enthusiasm and curiosity to concerns about ethical risks, data security, and the potential dehumanization of the physician–patient relationship (5).

Although there is growing consensus in the academic community regarding the integration of AI into medical education, this process remains limited in scope and often lacks practical implementation (6). Common barriers include a lack of faculty expertise, insufficient institutional infrastructure, curriculum overload, and competing educational priorities (7).

This gap limits the generalizability of existing findings and may hinder the development of globally relevant, equitable AI education strategies. Therefore, there is a pressing need for studies in developing countries and newly established institutions to inform context-appropriate curriculum reforms (8).

Notably, most published research on medical students’ perspectives on AI has been conducted in high-income countries and at well-established universities. Data from developing countries, particularly from newly established institutions, remain scarce (9). This represents a significant gap in the literature, as understanding student awareness, readiness, and educational needs in diverse settings is essential for creating equitable and inclusive AI training frameworks (10).

In this context, the present study, conducted among students of the Faculty of Medicine at Samsun University (a recently established public medical school located in a mid-sized city in the Black Sea region of Türkiye), offers a unique and valuable contribution. The findings of this study aimed to illuminate the AI-related knowledge, attitudes, awareness, and educational expectations of medical students outside major metropolitan centers. These insights may inform student-centered curriculum reforms and contribute to the development of a more balanced, inclusive national policy for AI integration in medical education. These results may inform local, regional, and national strategies to better prepare medical students for the digital transformation of healthcare.

## 2 Materials and methods

This descriptive and cross-sectional study was conducted among medical students at Samsun University Faculty of Medicine to evaluate their knowledge, awareness, attitudes, and educational expectations regarding AI in medical education. As Samsun University is a newly established institution, fifth- and sixth-year medical education had not yet been initiated at the time of the study (2024–2025 academic year).

### 2.1 Study design and setting

This study used a descriptive, cross-sectional survey design. Data were collected from medical students enrolled at Samsun University Faculty of Medicine, a recently established public medical school situated in Samsun, a mid-sized city in the Black Sea region of Türkiye. The study was conducted during the 2024–2025 academic year, focusing on the first through fourth years of the medical curriculum, as the fifth and sixth years had not yet been established at the institution.

### 2.2 Sample and participants

A structured questionnaire was initially distributed to all 235 actively enrolled medical students in the first, second, third, and fourth years at Samsun University Faculty of Medicine. Of these, 198 students voluntarily completed and submitted the questionnaire, yielding an 84.3% response rate.

As Samsun University is a newly established institution, the number of students enrolled in each academic year varied considerably due to staggered admissions over time. In particular, the inaugural cohort had a smaller class size, which naturally limited the maximum number of students that could be sampled from that group. To address this variability and ensure statistical comparability across academic years, a stratified random sampling method was used. Accordingly, an equal number of students (*n* = 30) were randomly selected from each academic year (first through fourth year), resulting in a final analytical sample of 120 participants. This approach aimed to ensure balanced representation, reduce sampling bias, and enable meaningful inter-year comparisons.

Additionally, participants were stratified based on gender to address potential gender bias, ensuring balanced representation. While participation was voluntary, which may introduce a degree of selection bias, random sampling from each academic year was implemented to mitigate concerns about the overall representativeness of the final analyzed dataset.

### 2.3 Data collection tool

Data were collected through a structured, self-administered questionnaire developed based on a comprehensive literature review on AI, digital health, and medical education (3, 6). Prior to distribution, the questionnaire underwent a content validation process involving expert evaluation by medical educators and pilot testing with a small group of medical students, consistent with established survey development guidelines (11). Feedback from experts and participants was used to refine the clarity, relevance, and comprehensiveness of the items (12). The survey was designed and administered electronically using Google Forms to facilitate efficient data collection and ensure anonymity. The survey link was distributed to students via secure online platforms and official class communication channels.

The questionnaire comprised four distinct sections, integrating both closed-ended and Likert-type scale questions to explore students’ perceptions and experiences.

Section A—demographic information (two items): This section gathered basic demographic data, specifically the participant’s current year of study in the medical program and gender.

Section B—technological literacy, interest in technology, and AI awareness (five items): This section aimed to assess students’ self-perceived proficiency in digital technologies (digital literacy), their general interest in technological innovations relevant to medical education (e.g., simulation systems and digital learning tools), their foundational knowledge of AI, their awareness of AI applications within medical education, and their engagement in independent learning activities related to AI. All items in this section were rated using a 5-point Likert scale, ranging from 1 (strongly disagree) to 5 (strongly agree).

Section C—attitudes toward AI and perceptions in medical education (10 items): This section investigated students’ beliefs and attitudes regarding the integration and impact of AI in various aspects of medical education. It covered perceptions of AI’s effectiveness in supporting simulation and interactive learning tools, its potential for personalizing education, its role in clinical decision support systems, its adaptability to diverse learning environments, its influence on student motivation, ethical considerations related to AI in medicine, and the necessity of integrating AI into the medical curriculum. Similar to Section B, responses were collected using a 5-point Likert scale (1 = strongly disagree and 5 = strongly agree).

Section D—student priorities and structured feedback (one item): This qualitative section allowed students to provide their primary recommendations for improving AI integration in medical education. Participants were presented with predefined options and asked to select the most critical area for improvement (3, 6, 13). The options included updating and enriching educational content; strengthening technological infrastructure (e.g., hardware, software, and Internet access); modernizing teaching methods and providing faculty training on AI; increasing integration into clinical practice (e.g., simulations and case-based learning); enhancing institutional and financial support; supporting student-centered projects (e.g., student research and hackathons); and promoting interdisciplinary collaboration with fields such as engineering and informatics.

### 2.4 Ethical approval

The study protocol and all associated materials were thoroughly reviewed and approved by the Samsun University Clinical Research Ethics Committee. The approval was granted on 18 March 2025, under Approval No: 2025/5/18 for non-interventional studies. Clinical trial number is not applicable. This observational survey study did not involve any clinical intervention or assignment.

All participants provided informed consent electronically before initiating the questionnaire, signifying their voluntary agreement to participate in the study. Strict measures were implemented to ensure the anonymity and confidentiality of all collected data. No personally identifiable information was requested or recorded from any participant.

### 2.5 Data analysis

Data collected from the online questionnaires were transferred and analyzed using IBM SPSS Statistics for Windows, Version 26.0 (IBM Corp., Armonk, NY, USA). Descriptive statistics, including frequencies and percentages, were calculated to summarize the demographic characteristics of the participants and the responses to the Likert-scale items in Sections B and C.

To identify potential associations and differences between categorical variables (e.g., gender and year of study) and students’ responses, the chi-squared test was used. For analyzing ordinal responses (e.g., Likert-scale data that are not normally distributed), the Kruskal–Wallis H-test was used to compare groups (e.g., across different academic years). A *p*-value of <0.05 was considered statistically significant for all analyses, indicating a less than 5% probability that the observed result occurred by chance. Responses to the predefined options in Section D were summarized descriptively to identify student priorities for improving AI integration in medical education.

## 3 Results

Key findings are presented in accordance with the questionnaire structure and analyzed with respect to demographic variables, year of study, and gender.

A total of 120 medical students from the first through fourth academic years at Samsun University Faculty of Medicine participated in the study, with 30 students randomly selected from each year. The analysis of responses is structured in alignment with the sections of the questionnaire: Section A (demographic information), Section B (technological literacy and AI awareness), Section C (attitudes toward AI and perceptions in medical education), and Section D (open-ended suggestions and feedback).

Descriptive statistics, including frequencies and percentages, were used to summarize the Likert-scale responses in Sections B and C. Group comparisons based on academic year and gender were performed using appropriate statistical tests. Responses from Section D were analyzed qualitatively to reflect students’ insights and priorities regarding the improvement of AI integration in medical education.

### 3.1 Section A: demographic information

The study sample consisted of 120 medical students from Samsun University Faculty of Medicine, with equal representation across the first, second, third, and fourth academic years (*n* = 30 per year). The distribution by gender was relatively balanced, with 62 students identifying as female (51.7%) and 58 as male (48.3%).

This even distribution across academic levels and gender provided a consistent basis for comparing AI-related awareness, attitudes, and perceptions among subgroups.

### 3.2 Section B: technological literacy, interest in technology, and awareness of AI

This section aims to evaluate medical students’ self-perceived competencies and attitudes toward digital technologies and AI. It focuses on three key dimensions: technological literacy, interest in educational technologies used in medical training, and the level of knowledge and awareness regarding AI and its applications in medical education. By exploring these areas, the study seeks to identify potential gaps in digital preparedness and engagement with AI among future healthcare professionals.

**Figure 1:**
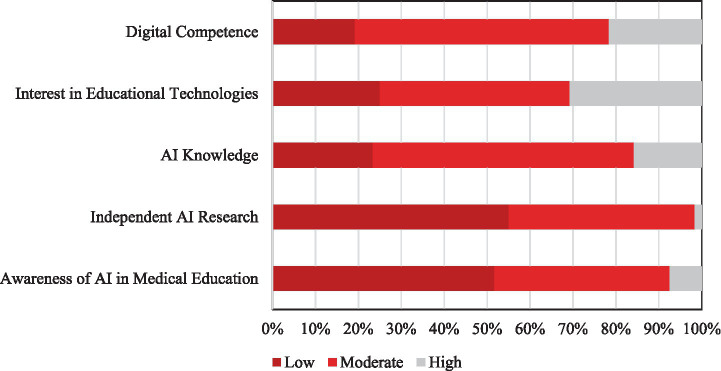
Distribution of medical students’ responses in digital competence, interest in educational technologies, AI knowledge, independent AI research, and AI awareness in medical education.

Students’ self-assessments revealed that the majority rated their digital competence at a moderate level (59.2%, *n* = 71). A smaller proportion considered themselves highly skilled, as 21.6% (*n* = 26) reported a high level, while 19.2% (*n* = 23) described their skills as low. These findings indicate that many students lack strong confidence in their technological literacy. A similar trend was observed for interest in educational technologies used in medical training. While 30.8% (*n* = 37) of students expressed high interest, nearly half (44.2%, *n* = 53) reported a moderate level, and 25.0% (*n* = 30) expressed low interest, suggesting a generally reserved attitude toward such tools. When asked about their knowledge of AI, the majority of students reported only a moderate understanding (60.8%, *n* = 73). In comparison, 15.8% (*n* = 19) of students considered themselves highly informed, whereas 23.3% (*n* = 28) reported low knowledge. Engagement with AI-related learning was also limited. While 43.3% (*n* = 52) of students indicated a moderate level of independent research, only 1.7% (*n* = 2) reported high engagement, and the majority (55.0%, *n* = 66) reported low or no activity. Similarly, awareness of AI applications in medical education was modest. Over half of the students (51.7%, *n* = 62) felt insufficiently informed (low), 40.8% (*n* = 49) reported a moderate level of awareness, and only 7.5% (*n* = 9) perceived themselves as highly informed. Overall, these results demonstrate that, while students generally place themselves at a moderate level across domains, relatively few report high competence, interest, or awareness, underscoring a clear need for curriculum-level improvements in medical education (Figure 1).

**Figure 2:**
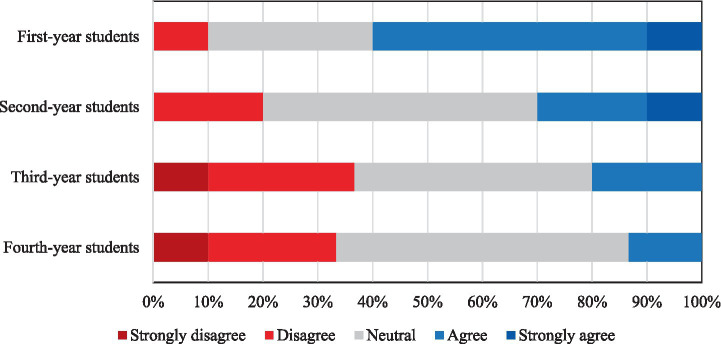
Students’ responses regarding their interest in technological innovations during medical education, presented by academic year.

A chi-square analysis revealed no statistically significant gender-based differences in technological literacy, interest in technology, AI knowledge, independent research, or awareness of AI applications (*p* > 0.05). However, comparisons by year of study showed a statistically significant difference only in interest in technology (*p* = 0.0180). No significant difference was found for personal AI research behavior (*p* = 0.2745). Post-hoc Mann–Whitney U-test analysis confirmed that interest in technology was significantly higher among first-year students than students in the other years (second year: *p* = 0.0253; third year: *p* = 0.0481; fourth year: *p* = 0.0053). No other pairwise differences reached statistical significance. Notably, while 60% of first-year students reported high interest in technology, this rate decreased sharply to 13.3% among fourth-year students (Figure 2). These findings suggest that younger students tend to be more interested in technology and AI-related topics, potentially due to earlier exposure to digital environments.

On the other hand, there were no significant differences across class levels in terms of perceived technological literacy, general AI knowledge, or awareness of educational applications (*p* > 0.05). This suggests that cognitive self-efficacy and foundational knowledge may remain stable across academic progression, whereas interest and motivation vary with age.

**Figure 3:**
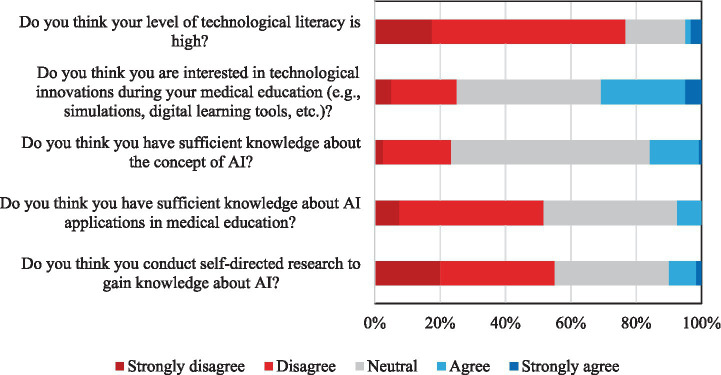
Students’ responses on technological literacy, interest in technology, and awareness of artificial intelligence.

These findings are visually summarized in Figure 3. The chart displays students’ responses regarding their technological literacy, interest in technological innovations, and awareness of AI. Notably, high rates of indecision and limited positive responses, particularly in items related to AI knowledge and personal research, support the conclusion that students generally lack confidence and sufficient engagement with AI-related content (Figure 3).

### 3.3 Section C: attitudes toward AI applications and perceptions of AI-supported medical education

Medical students generally exhibited highly positive attitudes regarding the integration of AI into their education and future clinical practice. A substantial majority of students (78.3%) expressed strong agreement that AI-supported interactive tools would significantly enhance clinical skill development and facilitate the transfer of theoretical knowledge into practical settings. Post-hoc analysis revealed that first-year students held significantly different perceptions on this matter compared to second-year (*p* = 0.0065) and third-year students (*p* = 0.0195). This enthusiasm also extended to AI’s potential for personalized learning, with the majority of students (79.2%) believing AI could effectively adapt to individual educational needs.

Students also demonstrated broad recognition of AI as a valuable tool for supporting clinical decision-making, with 80% expressing positive views on its role in clinical reasoning. Furthermore, there was widespread support (83.4%) for the adaptability of AI tools to diverse educational scenarios, indicating a desire for flexible and innovative learning environments. Notably, the perception that AI applications would enhance, rather than hinder, student–instructor interaction was prevalent, with 72.5% responding positively. The majority of students (69.2%) also anticipated that AI-supported educational materials would positively impact their learning motivation.

A significant proportion of students (65%) viewed AI-based educational approaches as more effective than traditional lecture and practical methods. Interestingly, post-hoc tests revealed significant differences in this perception between second-year and third-year students (*p* = 0.0238), as well as between third-year and fourth-year students (*p* = 0.0220). Crucially, students displayed a strong awareness of the importance of integrating ethical and legal dimensions of AI into the curriculum, with 78.3% agreeing that these aspects should be included. However, no statistically significant inter-year differences were found in attitudes toward the integration of ethical and legal issues in education (*p* > 0.05 for all pairwise comparisons). The value of multidisciplinary collaboration (e.g., with engineering and informatics) in enriching AI education content was also overwhelmingly recognized, garnering 84.1% agreement.

Overall, these findings reveal a strong, consistent student expectation for a more prominent role of AI and digital health technologies in medical education, with 82.5% advocating for broader curriculum integration. This widespread positive sentiment, consistent across genders and showing increased ethical awareness with academic seniority, presents a unique opportunity to accelerate comprehensive, student-centered curriculum reforms.

**Figure 4:**
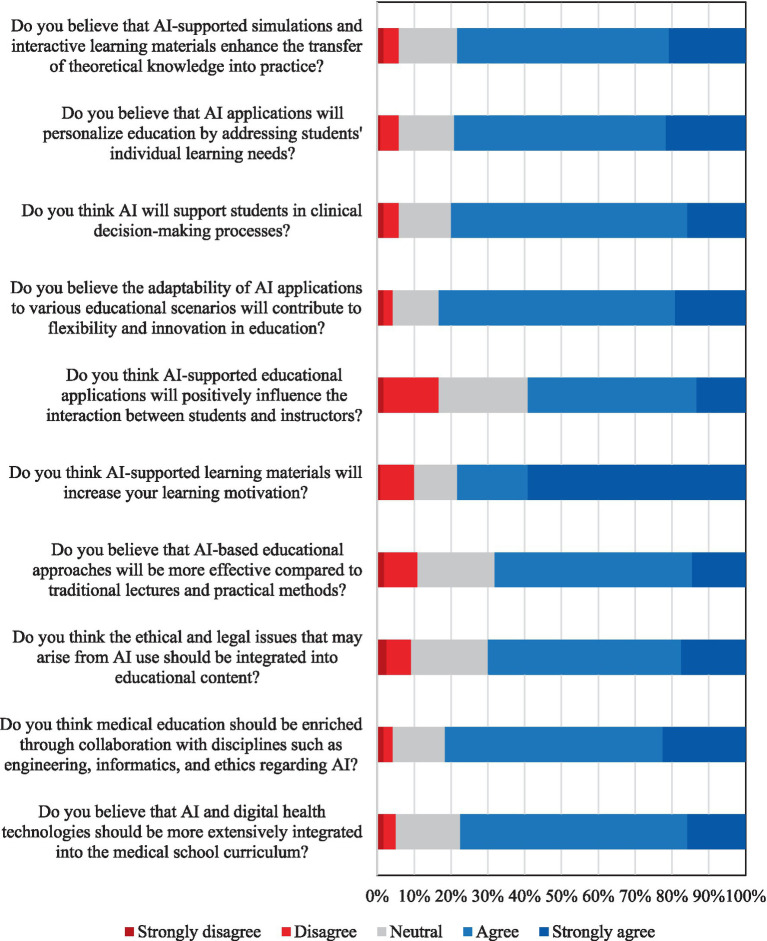
Students’ attitudes toward AI applications and perceptions of AI-supported medical education.

Figure 4 provides a visual summary of students’ attitudes toward AI applications and perceptions of AI-supported medical education. The distribution of responses consistently highlights generally positive perceptions across various domains, including the application of AI in knowledge transfer, personalized learning, student motivation, student–instructor interaction, and ethical integration within the curriculum. High rates of agreement and low levels of disagreement throughout these areas collectively reflect strong student support for the adoption of AI technologies in medical educational settings.

### 3.4 Section D: general suggestions and feedback

Participants were asked to indicate the most critical area for enhancing the integration of AI into medical education. The most frequently selected priority was increased integration into clinical practice (39.2%), followed by updating and enriching educational content (16.7%) and strengthening technological infrastructure (14.2%). Other preferences included modernizing teaching methods (10.8%), supporting student-centered projects (9.2%), promoting interdisciplinary collaboration (6.7%), and enhancing institutional and financial support (3.3%).

**Figure 5:**
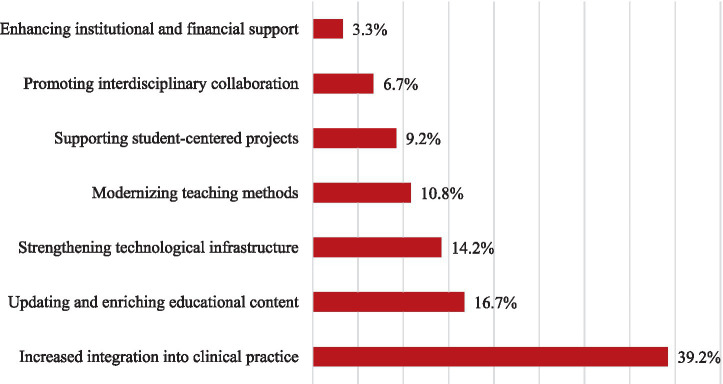
Distribution of students’ responses regarding key priorities for improving AI integration in medical education.

These findings demonstrate strong alignment with students’ previously reported attitudes, particularly their endorsement of AI-supported simulations and adaptive learning environments. The consistency across different sections of the survey supports the reliability of student perspectives as a valuable guide for future curriculum development. A detailed visual representation of these preferences is provided in Figure 5.

## 4 Discussion

This study aimed to assess the knowledge, awareness, attitudes, and educational expectations regarding AI among medical students at Samsun University, a newly established public medical school located in a mid-sized city in Türkiye. The findings provide a valuable perspective from a developing country, especially from an institution outside major metropolitan centers, thus contributing to a gap in the current literature, which predominantly focuses on high-income countries and well-established universities. These results may inform local, regional, and national strategies to better prepare medical students for the digital transformation of healthcare.

### 4.1 Technological literacy, interest in technology, and awareness of AI

One of the main findings of this study is that a large proportion of students placed themselves at a moderate level of digital competence; 59.2% of students reported moderate digital literacy, while 21.6% described themselves as highly skilled and 19.2% as poorly skilled. This is paralleled by AI-related knowledge distributions, where 60.8% of students described themselves as moderately knowledgeable, 15.8% as highly knowledgeable, and 23.3% as having low knowledge. Furthermore, engagement with independent AI learning was mostly modest: 43.3% of students reported moderate engagement, 1.7% reported high engagement, and 55.0% reported little or no activity, and awareness of AI applications in medical education was likewise modest (40.8% moderate, 7.5% high, and 51.7% low).

Overall, this pattern of incomplete preparedness aligns with findings from Saudi Arabia, where only 14.5% of students had received formal AI education and the vast majority felt insufficient regarding AI (14). Similarly, a study from Lebanon reported that 81% of students obtained their AI knowledge through media and social platforms and that insufficient formal curricula led to a lack of confidence and practical application (15). Another study from Türkiye also revealed that, despite high curiosity and interest, the vast majority of students felt inadequate in both theoretical and practical experience (16). These findings are also largely consistent with international trends. For example, Busch et al. reported in a global cross-sectional survey that students in medicine, dentistry, and veterinary science had limited AI knowledge and lacked formal AI courses, which contributed to a sense of limited preparedness for AI integration in their future careers (3). Other studies from developing countries such as Palestine have shown that over 74% of medical students had not received formal AI education and generally acquired their knowledge incidentally through media rather than structured curricula (4). In India, Kansal et al. found that, although students and doctors acknowledged the future importance of AI, the vast majority felt uninformed about AI applications (79.6%) and limitations (82.8%) (9). Thus, the results from Samsun University Faculty of Medicine underscore a widespread global challenge in AI literacy within medical education, particularly in developing countries. The low self-efficacy reported by this Turkish cohort may represent an initial barrier to AI integration; nevertheless, the global study by Busch et al. similarly highlights a general sense of unpreparedness (3).

The lack of comprehensive, applied, and systematic inclusion of AI and digital health education in medical curricula appears to be a fundamental cause of these deficiencies. In our study, interest in technological innovations was limited to 37.7% (sum of “interested” and “strongly interested” responses), while 53.1% of participants were neutral and 30.6% were uninterested. In Spain, Gualda-Gea et al. found that 69% of students had never used AI-based tools, and only 17% had moderate or advanced knowledge (17). Similar studies in the United States and the United Arab Emirates have shown that the majority of students only encountered AI technologies superficially and outside of formal courses (18). The finding that only 19.4% of students considered themselves knowledgeable in AI and 63.3% reported insufficient knowledge of AI applications in medical education further supports this situation. As with studies from Saudi Arabia, Lebanon, and the US, the multinational study by Ejaz et al. also demonstrates that, while students find AI potentially interesting, there are significant gaps in areas such as algorithm appraisal, clinical integration, and ethical application (13).

Another particularly interesting finding from our study is that interest in technology was highest among first-year students (60% reporting high interest) but dropped substantially by the fourth year (13.3% reporting high interest). This decline may reflect increased academic workload, lack of structured exposure to AI in the clinical years, or perceived irrelevance of AI to immediate clinical responsibilities. Targeted interventions are needed to sustain motivation across all years of study. A similar trend was observed in the multinational study by Ejaz et al. where students were more motivated and curious at the start of their education, but their interest in AI diminished notably during the clinical years under the weight of traditional curricula and practical demands (13). While this partially contrasts with Kansal et al.’s finding that Indian medical students showed more interest in AI learning than practicing doctors, it highlights the need to further investigate factors behind declining interest among more senior students (e.g., increased curriculum load, perceived irrelevance of AI to immediate clinical training) (9). This notable decline in interest among senior students could stem from multiple, intersecting factors. Increased curriculum load and preparation for national licensing exams in the clinical years may deprioritize topics perceived as peripheral to immediate clinical competency. The lack of structured, hands-on exposure to AI during hospital rotations may also reduce perceived relevance, as students focus on mastering traditional diagnostic and procedural skills. Furthermore, entrenched teaching methods and limited faculty engagement with AI topics may reinforce the perception that AI is tangential to medical practice. Similar patterns have been observed in multinational studies, where clinical immersion, while critical for professional identity formation, can inadvertently narrow academic curiosity toward emerging domains such as AI (3, 13). Addressing this issue may require integrating AI applications into clinical case discussions, simulation-based scenarios, and bedside teaching, ensuring that AI remains visible and relevant throughout the entire medical education continuum. In the Turkish context, additional cultural, infrastructural, and curricular factors may further shape these perceptions. The traditionally lecture-heavy and exam-focused learning culture, combined with limited digital infrastructure in some regions and rigid, content-dense curricula, can make it harder to accommodate emerging interdisciplinary topics like AI. These realities may partly explain the declining interest over time and underscore the need for reforms that adapt AI education to local educational norms while improving infrastructure and faculty readiness.

### 4.2 Attitudes toward AI applications and perceptions of AI-supported medical education

Despite the noted knowledge deficits, students in this study expressed overwhelmingly positive attitudes toward the integration of AI in medical education. A total of 78.3% of participants believed that interactive AI tools would enhance clinical skill development, 79.2% supported their use in personalized learning, and 80% saw them as beneficial for clinical decision-making processes. There was also a strong trend favoring AI’s potential to increase learning motivation (69.2% positive) and a belief that AI-based educational approaches could be more effective than traditional methods (65% positive). Students strongly supported the inclusion of ethical and legal aspects of AI in the curriculum (78.3% positive) and the promotion of multidisciplinary collaboration (84.1% positive). As a result, 82.5% of students advocated for broader integration of AI and digital health technologies into the medical curriculum. These positive attitudes were consistent across genders, and ethical awareness increased significantly with academic seniority (*p* = 0.0238).

These optimistic perceptions strongly align with global findings. Busch et al. reported that students worldwide have favorable attitudes toward AI in healthcare and a strong desire for more AI education (3). Studies from developing countries such as Palestine (4) and India (9) have similarly demonstrated a consensus on the need for AI in medical curricula. Research from Spain, the US, and the UAE has reinforced these findings, with students highlighting the advantages of AI-based education over traditional methods, including personalization, rapid feedback, and interactive learning (18). The enthusiasm observed among Samsun University Faculty of Medicine students suggests a high level of acceptance despite self-reported low knowledge, a dynamic that could be leveraged in implementing AI-focused educational reforms in Türkiye and similar developing countries. Leveraging these positive attitudes presents a strategic opportunity to accelerate curriculum reforms and implement student-driven, context-appropriate AI education models. The emphasis on ethics and multidisciplinary integration, also prominent in other countries, underscores the universal importance of these dimensions (19, 20).

### 4.3 General suggestions and feedback

Students’ recommendations for improving AI integration provide clear directives for educational reform. A total of 39.2% of students identified integration of AI into clinical practice as the most critical area, followed by updating and enriching educational content (16.7%), strengthening technological infrastructure (14.2%), and modernizing teaching methods (10.8%). These preferences align with students’ belief in the role of AI in bridging the theory-practice gap and are consistent with international calls for modernizing medical curricula to include AI (3, 6).

The literature repeatedly demonstrates that applied, hands-on education is more effective for motivating students and developing practical skills in AI (13, 20). To translate student enthusiasm into competence, institutions should move beyond theoretical discussions and implement actionable curricular changes. Specific examples include creating elective modules on topics such as “AI in Diagnostic Imaging” or “Ethics of Algorithmic Medicine,” developing simulation exercises where students use AI-powered decision support tools on virtual patient cases, and fostering interdisciplinary teaching models, such as joint “health hackathons” or projects with engineering and computer science departments. For Türkiye, a developing country seeking to expand its technological capacity in healthcare, these student-identified needs are particularly important. The emphasis on strengthening technological infrastructure is a critical first step, given the reported low digital competence and potential resource limitations. Therefore, addressing infrastructural deficiencies and investing in faculty development are foundational prerequisites for successfully launching such initiatives. These findings can inform student-centered curriculum reforms in Turkish medical schools and contribute to a more balanced and inclusive national policy for AI integration.

## 5 Strengths and contributions

This study provides valuable data from a relatively underexplored setting, a newly established medical school in a mid-sized Turkish city. This perspective, from a developing country and outside a major metropolitan center, helps fill a significant gap in the global understanding of medical students’ preparedness for the age of AI. The consistent positive attitudes despite low knowledge and the observed decline in technological interest with academic progression or year of study are noteworthy findings that warrant further investigation.

## 6 Limitations

Certain limitations must be acknowledged. As a single-center study, the findings may not be fully generalizable to all medical students in Türkiye or other developing countries. The cross-sectional design allows for the assessment of relationships, such as the observed decline in interest with academic advancement, but not causality. Additionally, self-reported data on knowledge and literacy may be subject to individual bias. Although thirty questionnaires from each academic year were randomly selected among volunteers for analysis, the initial overall response rate prior to random sampling was not detailed, which may introduce some selection bias. Moreover, as responses may over-represent students with higher intrinsic interest in digital health, generalizability to the entire student body should be interpreted cautiously.

## 7 Conclusion

Medical students at Samsun University display a strong willingness and positive attitudes toward the integration of AI into their education, despite currently limited knowledge and awareness. This study highlights the urgent need to develop and implement comprehensive AI curricula in medical schools, particularly in developing countries. Such curricula should move beyond theory to include actionable components, such as dedicated elective modules, hands-on simulation exercises with AI tools, and interdisciplinary teaching models that bring medical and engineering students together. These efforts must be supported by foundational investments in technological infrastructure and faculty development to ensure their success.

The gap between high enthusiasm and low preparedness represents a critical window of opportunity. Leveraging student interest through structured, engaging AI education can empower the next generation of physicians in Türkiye to use these technologies effectively and ethically. Bridging this gap requires urgent and applied curricular reforms grounded in interdisciplinary collaboration. Prioritizing the launch of pilot programs, such as a first-year “Introduction to Digital Health” seminar or a fourth-year “AI in Clinical Practice” simulation lab, can provide valuable local evidence and accelerate integration. Longitudinal studies are recommended to track changes in students’ knowledge and attitudes and to assess the long-term impact of these curricular interventions.

## Data Availability

The original contributions presented in the study are included in the article/supplementary material, further inquiries can be directed to the corresponding author.
